# Effect of bromocriptine on the severity of ovarian hyperstimulation syndrome and outcome in high responders undergoing assisted reproduction

**DOI:** 10.4103/0974-1208.69342

**Published:** 2010

**Authors:** Vinita Sherwal, Sonia Malik, Vandana Bhatia

**Affiliations:** Department of Reproductive Medicine, Southend IVF and Fertility Centre, Holy Angeles Hospital, Community Centre, Basant Lok, Vasant Vihar, New Delhi, India

**Keywords:** Assisted reproduction, bromocriptine, OHSS, controled ovarian stimulation

## Abstract

**CONTEXT:**

Ever since ovarian hyperstimulation syndrome (OHSS) was recognized as a clinical entity, various treatment modalities have been tried to prevent its occurrence and reduce its severity. Many recent studies have evaluated the role of dopamine agonists in reducing the incidence of OHSS.

**OBJECTIVES:**

To assess the effectiveness of dopamine agonist bromocriptine in reducing the incidence and severity of OHSS in patients undergoing assisted reproduction and its effect on pregnancy rates.

**SETTINGS AND DESIGN:**

It was a prospective study in which patients at high risk of developing OHSS undergoing assisted reproduction were recruited.

**MATERIALS AND METHODS:**

The study was carried out from August 2008 to August 2009 in patients undergoing assisted reproduction and included 40 patients at high risk for developing OHSS. Tablet bromocriptine 2.5 mg was prescribed for a period of 16 days starting from day of ovum pick up. Patients were analyzed on the basis of incidence of moderate and severe OHSS, timing of onset of OHSS (early/late), hospitalization rate, pregnancy rates and tolerability of medication and were compared with a historical group of high responders matched for age and BMI.

**STATISTICAL ANALYSIS:**

Student’s *t* test and proportion test were used.

**RESULTS:**

There was a significant reduction in the incidence of moderate OHSS (*P*=0.037), early OHSS (*P*=0.012) as well as the number of admissions (*P*=0.030). Average duration of hospitalization was also significantly reduced (*P*=0.036). In the study group, the incidence of clinically significant OHSS was 17.5% as compared to 40.9% in the control group. No difference was detected between the groups in clinical pregnancy rates (*P*=0.0054).

**CONCLUSION:**

Bromocriptine reduced the incidence and severity of clinically significant OHSS in high risk patients without affecting the pregnancy rates.

## INTRODUCTION

High responders are typically patients with an enhanced response to stimulation with ovulation inducing drugs. In assisted reproduction cycles (ART), they respond to stimulatory doses of gonadotrophins by forming an increased number of follicles, by rapidly rising and high serum estradiol (S.E2) levels on day of human chorionic gonadotrophin (hCG) trigger. High responders are usually patients who are young, with a low body mass index and patients with polycystic ovaries. The main risk in such patients undergoing ART is the development of ovarian hyperstimulation syndrome (OHSS) which in its severe form can be life threatening.[1]

The following preventive strategies have been used: cancelling the cycle, coasting, modifying the methods of ovulation triggering, administration of albumin or plasma expanders, cryopreservation of all embryos. Cycle cancellation by withholding the hCG trigger is the only foolproof method of preventing OHSS. However, it results in no pregnancies and oocytes. Some studies have also reported a low pregnancy rate in high responders. An increase in serum E_2_ levels seems to be detrimental to uterine receptivity without affecting embryo quality.[2]

Development of OHSS in women undergoing ART depends on the presence of functional ovaries and the administration of hCG.[3] It is thought that hCG increases vascular permeability leading to ascites and other complications of OHSS through vascular endothelial growth factor (VEGF) system, composed of ligands and receptors, which plays a pivotal role in the pathophysiology of OHSS.

Recently, dopamine agonists have been successfully used for patients at risk for OHSS and have reduced the severity of OHSS. They reverse increased vascular permeability in hyper stimulated animals by inhibition of vascular endothelial growth factor receptor-2 (VEGFR-2) phosphorylation.[4,5]

The present study has been designed to provide clinical confirmation of use of dopamine agonist bromocriptine as a new approach in decreasing the incidence and severity of OHSS in high responders and to analyze its effect, if any, on implantation rates. To this end, a prospective study was designed in which bromocriptine was employed in women at risk of OHSS after gonadotrophin administration. Bromocriptine was used instead of cabergoline because we have been routinely using bromocriptine in our unit for the treatment of hyperprolactinemia with excellent results. Also the fact that cabergoline has a longer half life and causes greater suppression of prolactin levels was a cause for concern as we know that prolactin is required for endometrial cell growth at low concentrations.[6]

## MATERIALS AND METHODS

This study was performed at our center from August 2008 to August 2009. Approval for carrying out the study was taken from the hospital ethics committee and informed consent was taken from patients enrolled in the study.

Out of 290 patients who underwent assisted reproduction, 40 patients were considered at risk of developing OHSS. Recruitment was done on the basis of presence of polycystic ovaries and/or previous history of OHSS, but patients were finally included in the study if there was retrieval of 20 or more oocytes and/or S.E2 levels were >3000 pg/ml on day of hCG trigger. We used a historical group of 44 patients from the period of August 2007 to August 2008 matched for age and body mass index (BMI) as the control group.

In all the patients, either a long protocol using an agonist or variable protocol with an antagonist was used. Agonists were started in the luteal phase of the menstrual cycle. Ovarian quiescence was confirmed by hormones and vaginal ultrasound following menstruation after which gonadotrophins were started. When antagonists were employed, patients received oral contraceptive pills during the cycle prior to that of ovarian stimulation. Gonadotrophin administration was started on second or third day of menstrual cycle and the antagonist was added at a dose of 0.25 mg/ day when the leading follicle reached a diameter of 14 mm. Ovaries were stimulated by recombinant follicle stimulating hormone (FSH) or human menopausal gonadotrophin (HMG) depending on patient requirement. Monitoring was done by transvaginal sonography (TVS) and serum estradiol (S.E2) levels. Recombinant hCG 250 microgram was administered when the mean diameter of at least two leading follicles reached 18 mm. Serum estradiol levels were measured on the day of hCG administration. Transvaginal oocyte retrieval was done under general anaesthesia 34 hours after the hCG injection. Embryo transfer was either done on second, third or fifth day. Two or three embryos were transferred. Serum beta hCG levels were done on day 16 after embryo transfer (ET). Luteal support was given with progesterone.

Patients at risk of OHSS were prescribed tablet bromocriptine 2.5 mg for rectal insertion starting on day of ovum pick up for a period of 16 days. They were asked to report any symptoms of OHSS like abdominal distension, nausea, vomiting, constipation, oliguria and breathing difficulties. Follow up was done on day 6, 9 and 16 after ET when TVS was done to see for ultrasound evidence of ascites.

Mild OHSS included abdominal distension, discomfort, mild nausea/vomiting and diarrhea.

Moderate OHSS was considered as mild features + ultrasonographic evidence of ascites (Perpendicular fluid pocket of >9cm^2^).

Diagnosis of severe OHSS required clinical evidence of ascites and/or hydrothorax and breathing difficulties or one of the following criteria: 1) increased blood viscosity i.e. hemoglobin at least 15 gm%, hematocrit at least 45%, or leucocyte count at least 20,000 per cubic millimeter. 2) coagulation abnormality 3) diminished renal perfusion and function (S.creatinine>1.2mg/dl) or 4) liver dysfunction, defined when transaminases (AST or ALT) are more than 40 u/ml.

Critical OHSS was diagnosed in the presence of tense ascites or large hydrothorax, hematocrit >55%, or leucocyte count>25000 per cubic millimeter, anuria, thromboembolism or acute respiratory distress syndrome.

Patients in study group were analyzed on the basis of incidence of moderate and severe OHSS (clinically significant OHSS), timing of onset of OHSS (early or late), hospitalization rate, requirement of paracentesis, pregnancy rates and any side effects of the medication and were compared with the historical control group.

### Statistical analysis

Statistical package for social science (SPSS) was used for statistical analysis. Categorical data were expressed as numbers and percentages and numerical data as mean±SD. Student’s *t* test and proportion test were used when appropriate. Significance was defined as <0.05.

## RESULTS

The study group had 40 patients while the historical arm control group had 44 patients. Table 1 compares the epidemiological data of the study and control group. The study and control group were similar with respect to age, BMI, mean units of FSH or HMG used, mean number of oocytes retrieved or peak estradiol levels on the day of trigger. Agonist cycles were employed in 29/40 patients (72.5%) in the study group and 34/44 patients (77.2%) in the control group, while antagonist cycles were used in 27.5% cases in study group as compared to 22.7% in controls. Out of 14 patients with OHSS in the study group, 5 patients underwent antagonist cycles.

**Table 1:** Epidemiological data and controlled ovarian hyperstimulation data

	Group A (study group)	Group B (control group)	*P* value
Total no. of patients	40	44	ns
Mean age (in years)	28.9±4.0	30.5±5.0	0.1115 (ns)
Mean BMI	22.5±3	21.6±2.5	0.1378 (ns)
Mean no. of oocytes retrieved	21.3±4.6	21.3±2.6	0.9847 (ns)
Mean S. E2 levels on day of trigger	3300±180	3250±190	0.2203 (ns)
Mean no. of days of stimulation	11.9 (9-15)	11.5 (8-14)	0.3375 (ns)
Agonist cycles	29 (72.5%)	34 (77.27%)	0.8011 (ns)
Antagonist cycles	11 (27.5%)	10 (22.72%)	0.8002 (ns)

Values expressed as mean±SD; ns- Not significant

The OHSS data is shown in [Table 2]. In the study group, out of 40 patients, 14 patients (35%) developed OHSS which was further characterized as mild in 7/40 patients (17.5%), moderate in 3/40 patients (7.5%), and severe in 4/40 patients (10%). The incidence of clinically significant OHSS was 7/40 patients (17.5%). In the control group, 24/44 (54.5%) patients developed OHSS out of which the number of patients having moderate OHSS was 12/44 (27.3%), a significantly higher number (*P*=0.0375) than those in the bromocriptine treated group, while 6/44 (13.6%) had severe OHSS leading to an incidence of 18/44 patients (40.9%) for clinically significant OHSS (*P*=0.0354). In the study group, only 3/14 patients had early OHSS while in the other group 14/24 cases were of early OHSS, a significant difference of 0.0125. Late OHSS cases were almost similar in the two groups. In the study group, all patients with mild and moderate OHSS were managed on outpatient basis and all the hospital admissions occurred in the severe OHSS group of 4/40 patients (10%), while in the control group there were 14/44 (31.8%) admissions, a significant difference of 0.0303. The average duration of hospitalization was also reduced in the study group (*P*=0.0365). The difference in paracentesis, hydrothorax and pericardial effusion was not significant. Outcome of ART is shown in [Table 3].

**Table 2:** OHSS incidence and outcome

	Group A (study group)	Group B (control group)	*P* value
OHSS cases	14/40	24/44	0.1155 (ns)
Mild OHSS	7 (17.5)	6 (13.6)	0.8480 (ns)
Moderate OHSS	3 (7.5)	12 (27.3)	0.0375 (sig)
Severe OHSS	4 (10)	6 (13.6)	0.8637 (ns)
Clinically significant OHSS	7 (17.5)	18 (40.9)	0.0354 (sig)
Clinical ascites	4 (10)	6 (13.6)	0.8637 (ns)
Paracentesis (no. of times)	6	14	0.1213 (ns)
Hydrothorax	2	4	0.7608 (ns)
Pericardial effusion	0	1	0.9711 (ns)
Hospital admission	4 (10)	14 (31.8)	0.0303 (sig)
Early OHSS (out of clinically significant OHSS)	3	14	0.0125 (sig)
Late OHSS (out of clinically significant OHSS)	4	4	0.8179 (ns)
Average duration of hospitalization	5.75±3.1	7.19±3.1	0.0365 (sig)

ns- Not significant; sig-significant, Figures in parentheses are in percentage

**Table 3:** Assisted reproduction outcome

	Group A (study group)	Group B (control group)	*P* value
Embryo transfer done in same cycle	40 (100)	24 (54.5)	<0.0001 (sig)
Embryo transfer deferred	None	20 (45.4)	<0.0001 (sig)
Clinical pregnancy	20/40 (50)	12/44 (27.2) Pregnancy in fresh cycles-8/24 (33.3%) Pregnancy in FET- 4/20 (20)	0.0543 (ns)
Delivered	9	8	0.8274 (ns)
Missed abortion	4	3	0.8928 (ns)
Mid trimester abortion	1	1	0.5128 (ns)
Ongoing pregnancy	6	None	
Multiple pregnancy	4/40 (10)	3/44 (6.8)	0.8928 (ns)
Multiple pregnancy in OHSS patients	1/7	2/18	0.9107 (ns)

ns- not significant; sig-significant, Figures in parentheses are in percentage

Embryo transfer was done in the same cycle in 100% patients in study group while in the control group ET was done in only 54.5% patients and was deferred in 20/44 (45.4%) cases (*P*<0.0001). In the study group, the pregnancy rate was 20/40 (50%). In the patients with OHSS, 9/14 (64.2%) were pregnant. In the control group, the pregnancy rate in fresh cycle transfers was 8/24 (33.3%) while in patients who underwent frozen embryo transfer it was 4/20 (20%). The cumulative pregnancy rate of control group was 12/44 (27.7%). The difference was not statistically significant (*P*=0.0543). The multiple pregnancy rate in study and control group was 10% and 6.6%, respectively.

In both the groups, patients having late OHSS were pregnant. One patient who developed severe OHSS in the study group had received injection hCG by mistake on day three after embryo transfer despite having free fluid.

The medication was well tolerated by all patients in the study group.

## DISCUSSION

The incidence of OHSS varies between treatments and patient groups and accurate estimates from the literature are difficult owing to the variety of classification schemes used historically. The majority of cases of severe OHSS are seen following *in vitro* fertilisation (IVF) treatment, but the syndrome can occur after any form of supraphysiological ovarian stimulation, including clomiphene and gonadotrophin ovulation induction. As many as 33% of IVF cycles have been reported to be associated with mild forms of OHSS. While these are often described as not being clinically significant, the severity of OHSS can worsen over time and even initially mild presentations should be kept under review. More severe OHSS has been reported in 3.1–8.0% of IVF cycles.[7] The incidence of OHSS is increased in young women, women with polycystic ovaries and in cycles where conception occurs, particularly multiple pregnancies. In a study by by Babayof *et al*. and Engmann L. *et al*. the incidence of clinically significant OHSS in patients at high risk for OHSS was 31%.[8,9]

A division of OHSS into ‘early’ and ‘late’, depending on the time of onset, may be useful in determining the prognosis. OHSS presenting within 3-7 days after the ovulatory dose of human chorionic gonadotrophin (hCG) is likely to reflect excessive ovarian response and the precipitating effect of exogenous hCG administered for final follicular maturation, whereas late onset OHSS is identified 12-17 days after ovulatory dose of hCG and reflects endogenous hCG stimulation from an early pregnancy. Late OHSS is frequently associated with multiple pregnancies.[10]

There is now a general consensus that women with ovaries primed with FSH/LH and subsequently exposed to hCG develop a clinical picture in which the ultimate pathophysiological step is increased vascular permeability.[11] Since hCG has no direct vasoactive properties,[12] investigations have aimed to detect the vasoactive substance responsible for this condition.

Albert *et al*.[13] have demonstrated that hCG stimulates the release of VEGF in human endothelial cells, which, in turn, acts in an autocrine manner to increase vascular permeability. VEGF over expression observed after hCG administration is related with this molecule’s high biological activity, which is 6–7 times that observed when LH binds to the same receptor. This is the consequence of the longer halflife of hCG and affinity for the common receptor.[14] VEGF induces vascular hyperpermeability by interacting with its VEGF receptor 2 (VEGFR-2).[15,16] Gómez *et al*.[17] showed that vascular permeability could be reduced by blocking VEGF receptor-2 and suggested that this approach could be used to prevent OHSS in humans.

But VEGF expression is important in the development of new capillaries from existing micro vessels prior to implantation, and improper vascularization of the endometrium may cause implantation failure and infertility. Accordingly, using VEGFR-2 inhibitors to treat or prevent OHSS during fertility treatment would also have the contrary indication of preventing pregnancy. It was demonstrated that increased vascular permeability could be reversed by using SU5416, a compound blocking the intracellular phosphorylation of VEGFR-2. But due to its side effect profile (thromboembolism, vomiting) and the possibility that it might interfere with early pregnancy development by blocking implantation-related ovarian and uterine angiogenesis, this drug cannot be used clinically to treat OHSS.[12,18]

Recently, it has been shown that pharmacological doses of dopamine, acting through the dopamine receptors (Dp-r2), can inhibit VEGF-mediated microvascular permeability, proliferation, and migration of endothelial cells *in vitro* by inducing endocytosis of VEGFR-2.[19] Prolactin secretion is a marker of Dp-r2 activity which is consistently reduced/ by administration of dopamine agonists. Dopamine agonists in current use are cabergoline, bromocriptine, pergolide, talipexole, ropinirole and pramipexole. Cabergoline is a long-acting ergot derivative agonist with a high affinity for D2 receptors. Bromocriptine is an ergot alkaloid dopamine receptor agonist. It is a strong D2 receptor agonist and a weak D1 receptor antagonist. It stimulates both pre and post-synaptic receptors. Gomez *et al*. in 2006 showed that dopamine agonist cabergoline (Cb2), when administered to immature rats at low doses simultaneously with hCG, prevented an increase in vascular permeability and did not affect angiogenesis.[4] Dopamine agonist cabergoline was used based on the finding that the gene coding for the enzyme tyrosine hydroxylase which is critical for dopamine production, was down regulated 8-fold in OHSS conditions. In this way, high VEGF expression and activity in OHSS seem to be associated with reduced dopamine production.[20]

Claudio Alvarez *et al* in 2007 also demonstrated that the effects of Cb2 were attributable to VEGFR-2 dephosphorylation and concluded that Cb2 may provide a new, specific and non-toxic approach to the treatment of OHSS, by which the increased vascular permeability, which is mediated by the VEGF/VEGR2 pathway, is blocked without altering angiogenesis.[5]

In our study, we used dopamine agonist bromocriptine in the dose of 2.5 mg daily. We found a beneficial effect of bromocriptine on the incidence of moderate OHSS which was significantly reduced. Bromocriptine also significantly reduced the incidence of early OHSS but failed to prevent late OHSS which was due to endogenous hCG stimulation from an early pregnancy. Probably, a higher dose of bromocriptine would be required in these cases to counter the effect of continuous hCG production by the developing pregnancy. Since the number of admissions in the study group was significantly lower than in the control group and the average duration of hospitalization was also significantly reduced, we can say that bromocriptine also reduced the severity of OHSS. Again this difference in admissions was observed in patients with moderate OHSS and not severe OHSS. In the control group, a number of patients with moderate OHSS had to be admitted due to persistent vomiting and significant abdominal discomfort, whereas in the study group all patients of moderate OHSS were managed on outpatient basis as they were more comfortable and there was earlier resolution of ultrasound evidence of ascites. Although the cumulative pregnancy rates in our series do not show a statistically significant difference, there is a marked difference in the fresh cycle transfers in the two groups. While in the study group fresh cycle transfer was done in all cases, in the control group 45.4% patients did not have a fresh transfer. This is important from the patient’s perspective who expects a fresh cycle transfer in the same sitting. It is also well known that pregnancy rates are better in fresh cycle transfers rather than in frozen embryo transfers. We did not find any association between multiple pregnancy and OHSS. Late OHSS cases always occurred in patients who conceived.

As bromocriptine was used rectally, the incidence of side effects was negligible.

Our results are comparable with a study carried out by Claudio Alvarez *et al* in 2007 in which they administered 0.5 mg cabergoline to a group of 35 women at risk of developing OHSS starting on day of hCG trigger. The total number of patients having moderate OHSS in their study group was seven (20%), whereas four (11.4%) were considered to have severe OHSS as against 43.8% having moderate OHSS and 18.8% having severe OHSS in the control group. Clinical pregnancy rate in their series was 48.6% whereas live birth rate per cycle was 40% and incidence of multiple pregnancy was 11.4%.^[21]^

## CONCLUSION

Our study found a beneficial effect of bromocriptine in reducing the incidence and severity of clinically significant OHSS in patients at high risk of developing OHSS without affecting the ART outcome.

The optimum dose of bromocriptine needs to be worked out in larger studies.

Further studies can be carried out to compare the efficacy of bromocriptine and cabergoline in preventing OHSS as also their role in treating established OHSS. Effect of bromocriptine on late OHSS by continuing bromocriptine in late luteal phase and early pregnancy can also be studied.
